# Editorial: Identifying clinically relevant transcriptional signatures and methylation profiles in the course, treatment and outcome of colorectal cancer

**DOI:** 10.3389/fonc.2024.1356765

**Published:** 2024-03-19

**Authors:** Pooneh Mokarram, Mozhdeh Zamani

**Affiliations:** Autophagy Research Center, Department of Biochemistry, Shiraz University of Medical Sciences, Shiraz, Iran

**Keywords:** CRC, epigenetic, genetic, immunotherapy, biomarkers

Colorectal Cancer (CRC) is currently one of the most aggressive neoplasms with increased incidence and mortality rates in the world. Conventionally, the key therapeutic policies in CRC include surgery, chemotherapy, radiotherapy, and targeted therapy. Among these therapeutic approaches, surgery is valid for patients with lesions in the early stages of diagnosis, and 20% of CRC patients miss surgery due to metastasis. Chemotherapy is also the main treatment option for CRC patients with metastatic states. Recently, targeted therapy has improved the process of colon cancer treatment, which has resulted in remarkable improvements in progression-free survival in metastatic CRC. Finding specific biomarkers for CRC screening and treatment is the prospect of scientific biomarker development in colorectal cancer-targeted therapy. In this regard, two related topics will be detected in this volume of Frontiers in oncology; the first is the role of triggering immune system by immunotherapy to prevent or treat cancer; and the second is novel genetic and epigenetic modifications as promising biomarkers for screening, prediction of prognosis, drug response, and metastasis in CRC. Aberrant DNA methylation and dysregulation of other epigenetic markers such as histone modification, MicroRNAs (miRNAs), Long non-coding RNAs (lincRNAs), etc. have been widely recognized as the most common characteristics of epigenetic alterations in CRC, having a role in CRC patients prognosis and treatment in the early stages (**Figure 1**). The most important and promising prognostic factor for CRC is the stage, the lower the stage of disease finding, the better the outcome. Emerging genetic and epigenetic biomarkers within the primary or metastatic tumor that are associated with prediction, recurrence, or favorable response have served as CRC biomarkers. However, in clinical decision making, heterogeneity is a vital factor affecting biomarker identification implementation and strategies. Molecular alterations cause CRC, which is a heterogeneous disease, such as gene methylation, and driver mutations, and is affected by the TME (Tumor Microenvironment), which causes infiltrates of immune cells through pro- and anti-tumor effects. Therefore, to develop effective immunotherapeutic strategies for CRC, it is important to study the interactions between the TME and the immune system (IS).

**Figure 1:**
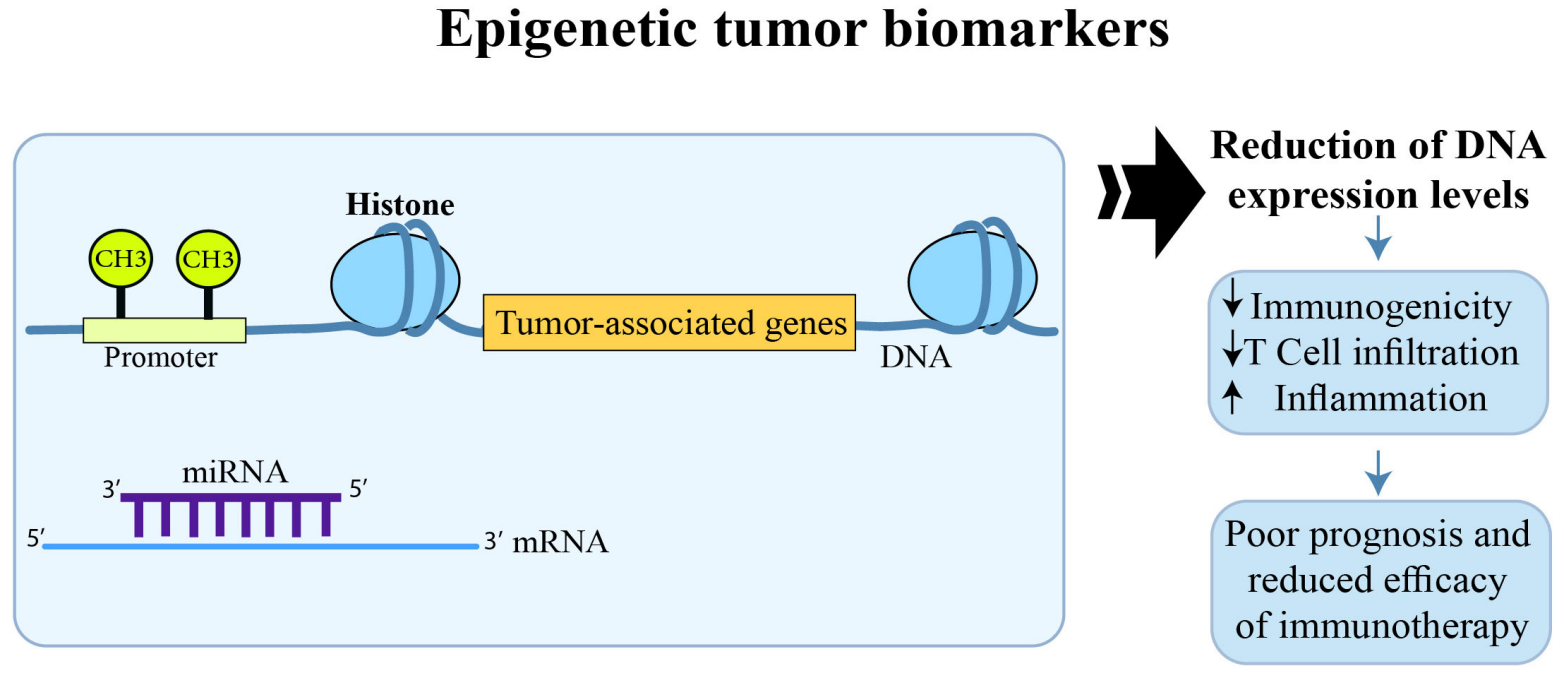
The effect of epigenetic modifications on CRC prognosis and response to immunotherapy.

Consistent changes in the expression of cell surface receptors, the presence of pro-inflammatory cytokines, chronic inflammation, and alterations in the downstream signaling pathways of Map kinase (MAPK) and TGF-β have been reported in the analysis of metastatic tumor tissue compared to the primary tumor (Holubekova et al.). Given the influence of the immune system on signal transduction pathways, it may be related to tumor-associated macrophage activity. The subsequent macrophage response supports the inflammatory and immune responses. The constant production of proinflammatory cytokines and chemokines leads to further changes in cellular pathways. Selection of tumor clones with blocked apoptosis, and the emergence of mutations cause mutation accumulation, cell cycle errors, and uncontrolled proliferation of tumor cells. On the other hand, genetic alterations such as KRAS mutations specifically affect adaptive and innate cytokine production and the immune system (Mei et al.). In addition, different infiltrations of six immune cells (macrophages, CD8+ T cells, neutrophils, B cells, dendritic cells, and CD4+ T cells) along with the change in expression of Peroxiredoxins (PRDXs) were clinically significant for the prognostic status of CRC which potentiate to be therapeutic targets or new markers for CRC (Zhou et al., Luo et al., Chen et al.). Meanwhile, the Cancer Genome Atlas (TCGA) cohort revealed differences in Transient Receptor Potential (TRP) channel expression between normal tissues and CRC. Furthermore, a poor prognosis was reported in CRC patients with low expression of Transient Receptor Potential Cation Channel Subfamily A Member 1 (TRPA1) and overexpression of Transient receptor potential cation channel subfamily M member 5 (TRPM5) and Transient Receptor Potential Cation Channel Subfamily V Member 4 (TRPV4), with these genes being considered as hub genes. Nevertheless, more detailed research is essential to fully realizing the role of TRP channels in cancer in order to plan novel, more precise, and valuable pharmacological tools. Moreover, activation of antitumor immunity occurred through TRP channels and upregulated M1 macrophages and CD4 + activated memory T cells. As one of the defining immune markers in CRC, CD103+CD39+ T cells may be a critical factor for antitumor immunity (Luo et al.). In these studies, the perspective of the immune infiltration landscape and personalized prognostic signatures have been identified.

In parallel to immunological alterations, novel genetic and epigenetic modifications have been introduced as promissing biomarkers for screening and prediction of prognosis and drug response in CRC. Authors have indicated that successful cancer screening approaches contribute to early detection, improved prognosis, and a reduction in cancer-related deaths. In this regard, higher colorectal cancer (CRC) detection rates have been achieved through a combination of syndecan 2 (SDC2) gene methylation testing and fecal immunochemical testing (FIT) using non-invasive stool samples (Zeng et al.). The addition of the serum carcinoembryonic antigen (CEA) test to the above-combined tests further increased the detection sensitivity. Therefore, stool-methylated SDC2 along with FIT and serum CEA can be considered a sensitive screening approach for the early detection of CRC (Zeng et al.). Hypermethylated glutamate ionotropic receptor AMPA type subunit 4 (GRIA4) has also been introduced as a potential tissue-specific biomarker for the early detection of CRC (Lukacova et al.).

Metastasis is also a critical factor leading to a remarkably low survival rate. Therefore, finding reliable metastasis biomarkers is essential for cancer management. Plasma hypermethylated GRIA4 has been considered a diagnostic target for non-invasive detection of metastasis (Lukacova et al.). On the other hand, tissue hypomethylated and overexpressed importin 5 (IPO5), upregulated hsa-miR-200, hsa-miR-135b-3p, and -5p, and also downregulated hsa-miR-548 were potential epigenetic biomarkers for colorectal cancer liver metastases (CRCLMs) (Horak et al.). Deregulated genes related to PI3K/AKT and WNT signaling pathways are potential biomarkers associated with CRCLMs. To differentiate Synchronous metastatic CRC (SmCRC) from Metachronous metastatic CRC (MmCRC) epigenetic modifications such as deregulated has-miR-1269-3p and hsa-miR-625-3p may be helpful (Horak et al.). The higher mutation burden in MmCRC and the significantly downregulated SMOC2 and PPP1R9A genes in SmCRC are other markers to distinguish SmCRC from MmCRC (Horak et al.).

Predicting tumor prognosis and drug response is particularly important for treatment planning and patient management. A novel risk model signature was constructed based on three genes of m5C regulator-mediated RNA methylation modification, including NOP2/Sun RNA methyltransferase 4 (NSUN4), NSUN7, and DNA methyltransferase 1 (DNMT1) (Zhang et al.). The prediction of prognosis and response to immunotherapy in rectal cancer patients is applicable using this novel signature. SPP1-CD44 and ALCAM-CD6 are two other immunological biomarkers of poor prognosis in rectal CRC (rCRC) patients (Jie et al.).

It could be concluded that epigenomic alterations in tumor cells are precise and promising predictors of the outcome of CRC patients. In addition, combination therapy with epigenetic drugs can achieve epigenetic changes to truly improve the efficacy response of cancer patients to targeted therapy which is applicable using novel biomarker signatures.

## Author contributions

PM: Writing – original draft. MZ: Writing – review & editing.

